# Asynchrony of wind and hydropower resources in Australia

**DOI:** 10.1038/s41598-017-08981-0

**Published:** 2017-08-18

**Authors:** Udaya Bhaskar Gunturu, Willow Hallgren

**Affiliations:** 1 Division of Physical Sciences and Engineering, King Abdullah University of Science and Technology, Thuwal, Saudi Arabia; 2 The MIT Joint Program on the Science and Policy of Global Change, Massachusetts Institute of Technology, Cambridge, 02139 MA USA; 3 Griffith Climate Change Response Program, Griffith University, Gold Coast Campus, Gold Coast, QLD 4222 Australia

## Abstract

Wind and hydropower together constitute nearly 80% of the renewable capacity in Australia and their resources are collocated. We show that wind and hydro generation capacity factors covary negatively at the interannual time scales. Thus, the technology diversity mitigates the variability of renewable power generation at the interannual scales. The asynchrony of wind and hydropower resources is explained by the differential impact of the two modes of the El Ni˜no Southern Oscillation – canonical and Modoki – on the wind and hydro resources. Also, the Modoki El Ni˜no and the Modoki La Ni˜na phases have greater impact. The seasonal impact patterns corroborate these results. As the proportion of wind power increases in Australia’s energy mix, this negative covariation has implications for storage capacity of excess wind generation at short time scales and for generation system adequacy at the longer time scales.

## Introduction

Wind and hydropower contribute up to 50% and 31%, respectively, of the installed renewable capacity in Australia^1^. While water availability is expected to be a key constraint due to climate change in the future^1^, abundant wind resources close to population centers in southern and eastern Australia^2^, and the maturity of the technology both facilitate the growth of wind energy. But it has generation characteristics very different from those of the conventional power resources. Most importantly, since wind resources are affected by atmospheric processes at different time scales, they are variable and intermittent. Grid integration of large-scale variable renewables is facilitated by availability of flexible resources in the power systems: largely dispatachable power plants, but to some lesser extent, electricity storage, demand management and interconnection to other power markets for easy power trade^3^. With large existing capacities of hydropower and due to its flexibility in ramping ability, hydropower forms an important flexible generation technology. Identifying this advantage, the International Energy Agency (IAE) formed a research and development Task (IEA Wind Task 24) titled ‘Integration of Wind and Hydropower Systems’ to investigate the opportunity for synergistic operation of integrated wind and hydropower system^4, 5^. Although this task force studied integration of wind energy on grids with hydro power resources, the constituent studies pertained to less than two years, and focussed on the short timescale variability.

The combination of several power generation technologies with different temporal variability provide generation system adequacy^6^ (GSA), which is defined as the existence of generating capacity to meet the peak load while accounting for the variabilities of generation and the load. The capacity value (contribution of the generator to meet generation system adequacy) of each of the generation technologies is central to the GSA. While the variability and uncertainty of wind power generation and its dependence on location reduce the capacity value of wind power^7^, it is also strongly affected by its ability to covary positively or negatively with other generation technologies. Since hydro and wind together form a substantial proportion of renewable generation in Australia, their covariation–positive or negative–is central to planning wind deployment and grid integration. If they covary positively (when both wind and hydro are at high levels), undispatched wind power can be stored in pumped hydro storage systems (PHSS). But when both are low due to positive covariation, substantial generation is lost. If they covary negatively, they compensate for each others loss.

A large fraction of the variability at the interannual time scales of geophysical variables is well understood as a consequence of the impact of the El Niño Southern Oscillation (ENSO)^8, 9^. Two modes of ENSO have been identified–canonical ENSO^8^ (CE) and Modoki ENSO (ME)^10^. Both the modes have their positive (El Niño) and negative (La Niña) phases. The positive phases of CE and ME are the canonical El Niño (C-EN) and the Modoki El Niño (M-EN). The negative phases are the canonical La Niña (C-LN) and the Modoki La Niña (M-LN). Because the two modes are independent, their impacts are likely to be different in different parts of the world. In this study, the covariation of wind and hydro power in Australia at interannual scales and the contributing impacts of ENSO are assessed. Since both wind and hydro resources are influenced by ENSO, the covariation of these two resources in Australia is a result of the differential impact on each of them.

## Results

### Interannual variation of generation capacity factors

**Figure 1:**
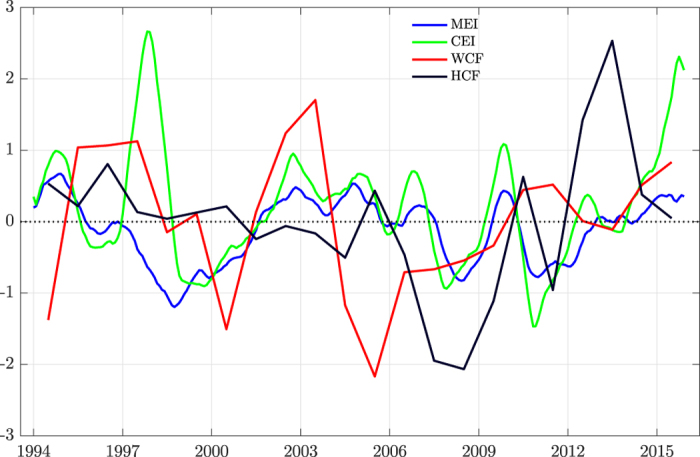
Capacity factors and ENSO indices: 12 point moving averaged canonical (CEI) and Modoki (MEI) ENSO indices and standardized capacity factors of wind (WCF) and hydropower (HCF). The unit along the y-axis is standard deviation.

The annual wind and hydro power generation^11^ and the respective installed capacities^12^ have been used to compute the annual capacity factors of wind (WCF) and hydro (HCF) power in Australia (described further in ‘Data and Analysis’ in the supplementary information). Figure 1 shows the interannual variation of the WCF and HCF for the whole of Australia. Since almost all of the wind installations in Australia are in southeastern Australia (SE South Australia, Victoria, Tasmania and New South Wales), WCF effectively corresponds to this region. The standardized detrended indices of CE (CEI) and ME (MEI) that represent the intensity of CE and ME respectively have also been plotted. The WCF corresponds well with the MEI. Although WCF varies less between 2007 and 2014 due to an absence of strong CE or ME events compared to the previous years, since the installed wind power capacity increased exponentially during this period, the variation of effective wind power generated is very large. There are interannual ramps of more than 50% and they resulted in large interannual variation in wind power generation. While some peaks and troughs of WCF correspond to the peaks and troughs of CEI or MEI, the relationship breaks down during some years. Thus, it is clear that the relationship is more complex than just covariation. Further, the covariation of annual WCF and HCF is not very clear before 2000 as the installed wind capacity was very low (2–10 MW). But as the installed capacity increased, there is an approximate negative covariation. It is to be noted that the variability of WCF decreased after 2009. But as the installed capacity and hence the generation increased exponentially, the variability of the actual generation is high. Also, the variability of HCF increased after 2004. Thus, the negative covariation persisted throughout. It is to be noted that the variability of WCF decreased after 2009 as the wind power deployment occurs in locations with lower resource quality (abundance). But as the installed capacity and hence generation increased almost exponentially, the variability of the actual generation is high. To understand the interannual variability of WCF and its covariation with HCF, the impact of CE and ME on the wind resource in Australia is examined in greater detail in the subsequent paragraphs.

### Impact of the Canonical and Modoki El Niño on the wind resource

**Figure 2:**
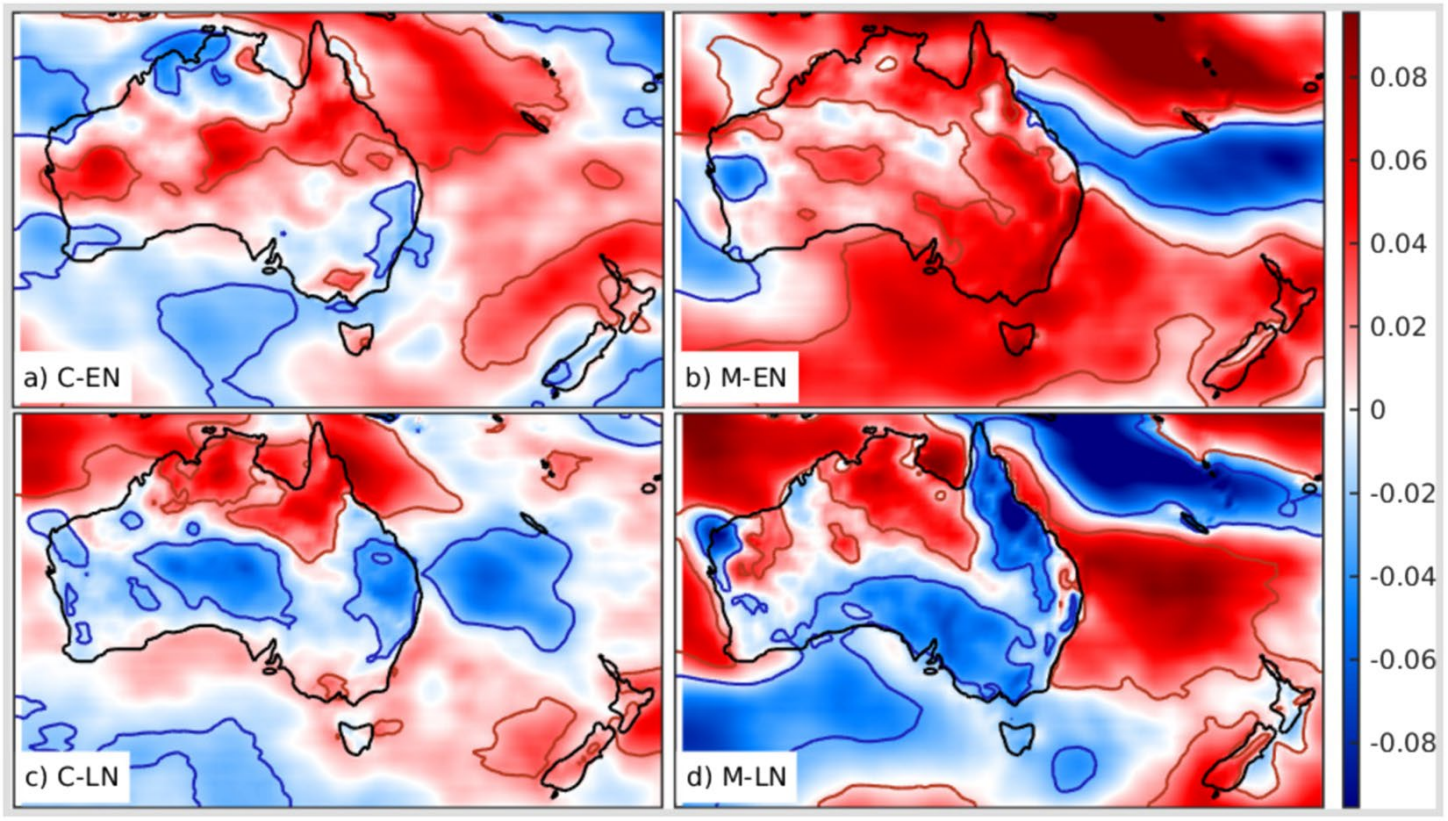
Composites of WPD anomalies: The mean standardized anomalies of WPD corresponding to (**a**) C-EN, (**b**) M-EN, (**c**) C-LN, and (**d**) M-LN. Red (blue) contours show regions with statistically significant increase (decrease) at 90% significance level. The unit along the colorbar is standard deviation. The figure including the map and all the text elements has been plotted in MATLAB version R2016a. (http://www.mathworks.com/products/matlab/).

The C-EN composite of the wind power density (WPD) anomaly from its long term mean has been constructed by averaging the WPD anomalies corresponding to the days categorized as C-EN. Similar composites have been constructed for M-EN, C-LN, and M-LN. The composites of WPD anomalies (Fig. 2) show that there is a significant increase of the resource during M-EN and a decrease during M-LN. During M-LN, there is a lower resource in SA, Victoria, Tasmania and coastal NSW. However, there is not much impact of C-EN and C-LN apart from a decrease in the resource in the east coastal NSW. During neutral CE and ME the mean anomalies of the WPD are very low (Figure S1). The wind resource in the different regions of Australia is affected by the cycle of seasons differently. But overall, Australia has stronger winds during winter and spring. Further, CE is phase-locked to the southern hemisphere (SH) summer; ME is less so. Thus, it is imperative to elucidate the impact of CE and ME on the wind resource in warm (October-March) and cool (April-September) seasons separately.

**Figure 3:**
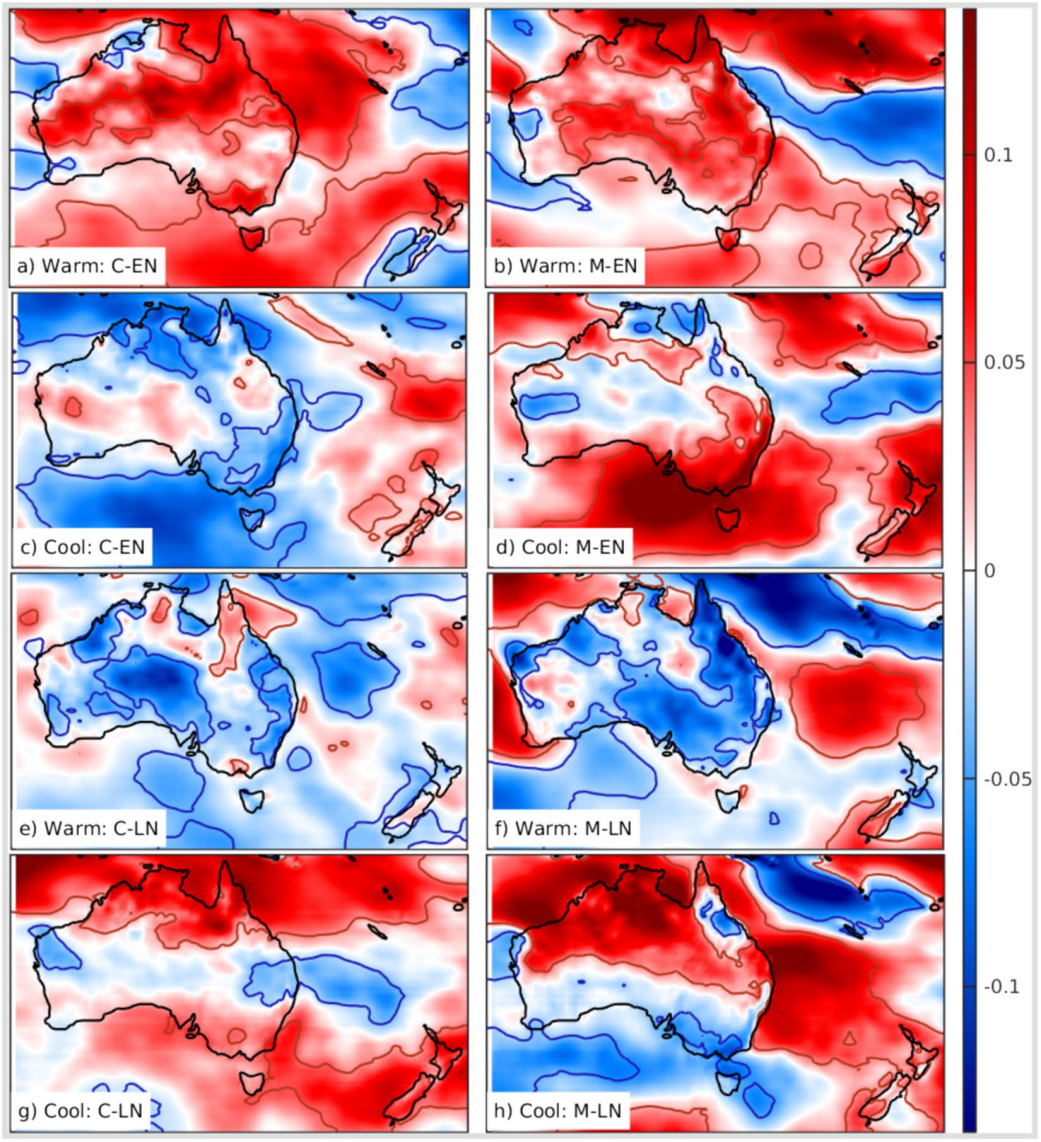
Seasonal composites of WPD anomalies: The mean standardized anomalies of WPD. The season and ENSO mode and phase are shown in the lower left corner of each panel. Red (blue) contours show regions with statistically significant increase (decrease) at 90% significance level. The unit along the colorbar is standard deviation. The figure including the map and all the text elements has been plotted in MATLAB version R2016a. (http://www.mathworks.com/products/matlab/).

When divided into warm and cool seasons, distinct patterns of the impact on the WPD are revealed as shown in Fig. 3. The impacts of C-EN on the WPD in the cool and warm seasons are almost opposite. So, when both seasons are combined, these opposite impacts cancel each other as in Fig. 1. The effect of strong M-EN events in the two seasons is largely the same, that is to increase the wind resource but in different areas of southern, southeastern and eastern Australia. Further, the magnitude of the impact is also large in the opposite seasons. These composites show the mean seasonal impact of CE and ME. The actual instantaneous impact depends on the instantaneous strength of C-EN, M-EN, C-LN and M-LN events. Thus, it can be inferred that the interannual variability in the WCF in Fig. 1 can be explained by a linear combination of the impacts of CE and ME while taking the season of the impact into consideration. Figure S2 shows that the mean anomalies are very low during neutral CE and ME in both the seasons.

## Discussion and Conclusions

The most important results of the present study are: (a) the wind and hydro power generation covary oppositely in Australia (b) the M-EN phase enhances the wind resource in regions of Australia with abundant resource except small areas in western Australia, and (c) both C-LN and M-LN decrease the resource in most of Australia.

Southeastern and southern Australia is rich in renewable resources; and Tasmania (87%) and South Australia (40%) depend on renewable energy for power generation. Hydro and wind power constitute nearly 46% and 31% of the renewable generation in Australia and are largely collocated^1^. Most of the hydropower is generated in eastern Australia and Tasmania, and most of the wind resource in south and southeastern Australia. Because of high interannual variability in rainfall, hydropower is also highly variable at those scales and causes resource inadequacy during drought periods. The impact of both C-EN and M-EN in Australia is to lower the rainfall and hence the stream flow^13^, with M-EN having the stronger impact^14, 15^. The Millennial drought due to these impacts resulted in a huge fall in hydropower generation in Australia^1^.

Apart from providing elucidation of the impacts of CE and ME on the mean wind resource in different parts of Australia, the statistically significant impacts shown here can allow the forecasting of changes in the mean resource. The Australian Bureau of Meteorology^16^ monitors the development, evolution and decay of CE and ME events routinely. This information can be used in conjunction with the results of this study to forecast the impact on the wind resource in a region several months ahead.

The most significant inference of this study is the asynchrony between hydro and wind power resource in Australia. CE and ME impact the hydro and wind resource such that they have a generally opposite variation. The exceptions can be explained from the seasonal patterns of the impacts. Thus, hydro and wind power compensate the resource inadequacy of each other at interannual scales. Hydropower capacity has almost peaked in Australia as most of the resources have been dammed. But since wind deployment is growing at a fast rate, and is expected to reach 12.1% of the national energy mix by 2030^1^, this compensation of the two mature technologies is very significant and has two critical consequences. At longer timescales wind and hydropower compensate the resource inadequacy of each other. But at shorter timescales the excess wind power cannot be stored in PHSS as a hydrological drought limits the availability of water in PHSS. A power system-based study^5^ of the Tasmanian power system showed that it could not integrate all the wind power from large-scale generation due to coincident wind and hydro generation. Also, the storage capacity of wind generation was negatively impacted as wind penetration increased. It is to be noted that this study was based on hydro inflow data prior to 2005^4^. As this study shows, due to the increasing impact of the ME, the wind and hydropower are oppositely impacted.

Non-availability of wind power generation data at monthly or higher frequency is a limitation of this study. Although inter-annual variability can be ascertained with this dataset, monthly or finer scale data can elucidate interannual variability of mean seasonal wind power generation. As discussed earlier, wind power generation is strongly dependent on season. Most regions in the world have high wind power density and hence higher wind power generation during winter than that in summer. Other assumptions in the construction of the wind power density like the coarse resolution of the MERRA reanalysis and neutral stability of the atmospheric boundary layer are discussed in a previous study^2^. Notwithstanding the limitations of the wind power generation data, as has been shown, the negative covariation of the resources–wind and hydro–is very robust.

Australia has 1.5 GW of PHSS^1^ in the southeast of the country which is critical to store excess wind power and mitigate its intermittency. Resource adequacy problems are likely to be exacerbated at the higher future proportion of wind power^17, 18^. While there are a range of other factors like the withdrawal of coal power, the high reliance on gas, limited import capacity and concentrated generator ownership, high levels of wind in the energy mix is the most important contributor to resource inadequacy, high power prices and price volatility in Australia, particularly in South Australia^18^. Wind variability, due to different modes of ENSO, and the synergy between wind and hydropower, which have been assessed in this study, are likely among the important causes of intermittency and resource inadequacy, and will need to be addressed if the potential of this combination of renewable energy sources is to be realised.

The frequency and intensity of M-EN events have increased since the late 1990s^19^, causing extended durations of hydrological droughts. As a result, the hydropower decreased by 4.2% per year from 1999 to 2008^1^. The frequency of C-EN is expected to increase due to anthropogenic climate change^20^ and that of M-EN five times the frequency of C-EN^21^. Assessments of the impacts of climate change project southeastern Australia to be drier, with higher evaporation and reduced rainfall^22, 23^. Therefore, the generation from existing hydropower generators is expected to decrease by 18%^24^. Further, due to the rise of other energy technologies (wind, solar, gas etc), the proportion of hydropower is expected to fall from 4.5% of the total electricity generation to 3.5% by 2030^1^ without taking the impact of climate change into consideration. As wind power deployment grows, constraints on land use and availability of transmission capacity may limit further development to South Australia, Victoria, Tasmania and New South Wales^1^. Harvesting of potential offshore resources may also occur in the same region. Thus, the variability caused by wind droughts due to the impact of CE and ME is a central factor to be considered for ensuring resource adequacy. Further addition of wind power to the system is likely to enhance resource adequacy^25^, but, at shorter time scales, mitigation of the variability of wind power at such large penetration levels requires increased storage like PHSS. Also, as wind power penetration level increases, the capacity value of wind is expected to rapidly decrease in the Australian National Electricity Market^26^.

Since the variability and intermittency of wind power are inherently connected to the prevailing weather, the level of penetration will depend on efficient grid management. A salient strategy is to install sufficient capacity to manage the fluctuations. At small levels of deployment the wind power generation is treated as a small fluctuation in the demand. Although hydropower is also variable, the variability is at seasonal scale and beyond. As wind overtakes hydro as the leading renewable power resource in Australia^1^, grid level storage, back-up generation (for instance, using gas), and robust forecasting assume greater importance to mitigate variability, particularly when the most flexible generation–hydro–is deficient. Further, it is imperative to assess the different resources at different sites so that technology and geographical spread of renewable generation results in the least variable aggregate power that fulfils the demand^3^. An assessment of the flexibility in the power system provided by dispatachable generation, interconnection, storage and demand management can inform the maximum level of penetration of renewable energy. Understanding the variability and intermittency of the constituent components, largely wind and hydro, at different time scales is an essential component of such flexibility assessment.

## Data and Methods

### Data

The following datasets have been used for this study.

1. Annual installed capacities of wind and hydropower in Australia from 1994 to 2015 from the OECD-Net capacity of Renewables dataset^12^ by the International Energy Agency (IEA). This dataset contains the installed capacities of different energy technologies data collected by the national statistics offices, government agencies, industry organizations and other public bodies of the member countries.
2. Annual generated wind and hydropower from 1994 to 2014 in Australia from the OECD-Renewable Supply and Consumption dataset^11^ by the IEA. This dataset contains the energy supply and consumption data collected by the national statistics offices, government agencies, industry organizations and other public bodies of the member countries.
3. Monthly values of Multivariate El Nino Index^27^, which shows the strength of the canonical ENSO, have been taken from the NOAA website^28^. This is referred to as CEI in this study. The cited ref. 27 also describes the methodology of construction of these indices from the sea surface temperatures in the Pacific basin.
4. Monthly values of El Nino Modoki Indexi^10^ (denoted as MEI in this study) that show the strength of the El Nino Modoki oscillation have been taken from the Japan Agency for Marine-Earth Science and Technology^29^ dataset. The cited ref. 10 also describes the methodology of construction of these indices from the sea surface temperatures in the Pacific basin.
5. Conventionally wind speed has been used as a metric to describe wind power resource. But wind power density (WPD), defined as:

1 $$WPD=\frac{1}{2}\rho {V}^{3}$$

is an accurate measure of the energy content of wind as this is a measure of the thrust acting on the turbine blades. WPD is technically defined as the kinetic energy contained in a unit cross-sectional area normal to the wind. Here, *ρ* is air density and *V* is the wind speed.

Global observations from different platforms (satellites, surface observations, buoys, weather balloons etc) are assimilated into a General Circulation Model to obtain a physically and dynamically consistent state of the atmosphere. This method is generally called Retrospective Analysis (Reanalysis). By this process, a ‘physically and dynamically consistent’ data is generated even where there are no observations. Since the observations are assimilated every 6 hours into the model, the dataset generated by this ‘data assimilation’ are considered robust. They are particularly useful where there are no observations and where the record lengths of the observations are short or intermittent. We use a reanalysis dataset called Modern Era Retrospective Analysis for Research and Applications (MERRA) developed by NASA’s Global Modeling and Assimilation Office^30^ by assimilating the global observations into the GEOS-5 model. From this dataset, we use the atmospheric boundary layer parameters: surface roughness length, friction velocity, displacement height and air density.

Using these variables, we construct the wind speed at 80 m hub height using the expression:

2 $$V=(\frac{{u}^{\ast }}{k})[\frac{log(z-d)}{{z}_{0}}]$$

where, *V* = the wind speeed at 80 m hub height, u* = friction velocity, *k* = von Karman constant, *z* = hub height (80 m), *d* = displacement height, and *z*_0_ = is the roughness length of the atmospheric boundary layer. The constructed wind speed in Australia^2^ has been validated by comparing with wind atlas published by the Australia Government Department (AGD) of the Environment, Water, Heritage, and the Arts. This atlas was generated by WindLab for the AGD using weather observations from station data taken from Australia Bureau of Meteorology for the years 1995–2005, and supplemented with commercially generated meteorological datasets. Our wind resource construction has also been validated using publicly available maps of the location of wind farms in South Australia and New South Wales. This method^2, 31^ of construction of wind power resource improves upon previous constructions of the wind resource that suffered from coarse spatial resolution, sparse and uneven spatial coverage, shortness of record length, and/or low temporal resolution^32^.

The data used for this study is an updated version of the one used in a previous study^2^. Nearly 37 years of hourly 0.5 × 0.67 (latitude x longitude) resolution MERRA data (from 0030 on January 1st, 1979 to 2330 on 30th June, 2015) was used to reconstruct the wind field at 80 m (above the ground) turbine hub height. The 80 m hub height is the standard without technology developments. Wind speed and then wind power density were computed at this height. The domain considered for our study spans the entire Australian continent including Tasmania and New Zealand, between 10S and 45S degrees latitudes and 110E and 155E longitudes.

## Analysis

### Capacity factor

Capacity factor (CF) for an energy technology is defined as the ratio of the energy generated by that technology during a year to the maximum expected energy generated in a year.

3 $$CF=\frac{{\rm{Generation}}\,{\rm{in}}\,{\rm{a}}\,{\rm{year}}\,{\rm{in}}\,{\rm{MWh}}}{{\rm{Installed}}\,{\rm{capacity}}\,{\rm{in}}\,{\rm{MW}}\ast {\rm{NoHy}}}$$

where NoHy is the number of hours in the year. WCF and HCF have been computed using this expression. Since CF is independent of the technology deployed (for instance, the turbine for wind power) for harvesting the energy resource, it largely captures the variability of the resource.

### Standardization of the variables

Comparison of quantities is rendered less robust by the differences in the distributions of the quantities. Standardization enables us to bring the different quantities to one common distribution so that they can be compared robustly. For robust comparison of different quantities, the different quantities (HCF, WCF, and WPD anomalies) are standardized as follows:

4 $${x}_{i}^{z}=\frac{{x}_{i}-{\mu }_{x}}{{\sigma }_{x}}$$

where ${x}_{i}^{z}=$ the standard score of *x*_*i*_ in the distribution of *x*_*i*_, *μ*_*x*_ is the mean of *x*_*i*_, and *σ*_*x*_ is the standard deviation of *x*_*i*_. The absolute value of ${x}_{i}^{z}$ shows the distance of *x*_*i*_ from *μ*_*x*_ in number of standard deviations. The unit of a standardized variable is standard deviation.

### Composites of WPD anomalies

The anomalies of the WPD at each grid point were computed by subtracting the climatological mean of the hourly WPD from the hourly WPD values. Daily mean values of the anomalies were computed by averaging the hourly values in each day.

The monthly values of CEI have been used to characterize each month as C-EN, C-LN or C-NU based on the following criterion:

5 $$mm=\{\begin{array}{ll}C-EN & {\rm{CEI}}\ge 80{\rm{th}}\,{\rm{percentile}}\,{\rm{of}}\,{\rm{CEI}}\\ C-LN & {\rm{CEI}}\le 20{\rm{th}}\,{\rm{percentile}}\,{\rm{of}}\,{\rm{CEI}}\\ C-NU & {\rm{otherwise}}\end{array}$$

Similarly, each month is characterized as M-EN, M-LN, or M-NU based on the following criterion:

6 $$mm=\{\begin{array}{ll}M-EN & {\rm{MEI}}\ge 80{\rm{th}}\,{\rm{percentile}}\,{\rm{of}}\,{\rm{MEI}}\\ M-LN & {\rm{MEI}}\le 20{\rm{th}}\,{\rm{percentile}}\,{\rm{of}}\,{\rm{MEI}}\\ M-NU & {\rm{otherwise}}\end{array}$$

The WPD anomalies for all the days of the months corresponding to C-EN are averaged to get the composite of WPD anomalies corresponding to C-EN. Similarly, the composites of WPD corresponding to C-LN, C-NU, M-EN, and M-NU are computed for all the points in the domain.

At each gridpoint, 95% of C-EN anomalies and 95% of C-NU anomalies are resampled 10000 times randomly and Mann-Whitney rank-sum test has been used to test the following hypotheses at 90% significance level:

- Null(*H*_0_): The probability of a C-EN anomaly exceeding a C-NU anomaly is 0.5.
- Alternative(*H*_1_): The probability of a C-EN anomaly exceeding a C-NU anomaly is greater than 0.5.
  Similarly, the Mann-Whitney test has been applied to test the hypotheses:
- Null(*H*_0_): The probability of a C-EN anomaly being less than a C-NU anomaly is 0.5.
- Alternative(*H*_1_): The probability of a C-EN anomaly being less than a C-NU anomaly is greater than 0.5.

Red and blue contours are used to delineate regions statistically significant positive and negative anomalies respectively. Similarly, statistical significance is estimated for C-LN, M-EN and M-LN.

### Seasonal composites of WPD anomalies

CE and WPD have strong seasonal pattern: CE is phase-locked to SH summer and the wind resource is stronger in SH winter. Thus, it is imperative to assess the impact on cool (April-September) and warm (October-March) seasons separately. Therefore, the constructed WPD dataset is segregated into cool (winter) and warm(summer) WPD datasets and the above analysis is applied on these two seasonal wind resource datasets.

## Electronic supplementary material

Supplementary information
