# Supplementary material for: Asynchrony of wind and hydropower resources in Australia
Source: Sci Rep. 2017 Aug 18;7:8818. doi: 10.1038/s41598-017-08981-0 (PMC5562748; doi:10.1038/s41598-017-08981-0)
Supplement: Supplementary file 1 — Supplementary information [file 41598_2017_8981_MOESM1_ESM.pdf]

Asynchrony of wind and hydro power resources in Australia: Supplementary information

Udaya Bhaskar Gunturu[1,2] and Willow Hallgren[3]

[1]The MIT Joint Program on the Science and Policy of Global Change, Massachusetts Institute of Technology, Cambridge, MA 02139, USA

[2]King Abdullah University of Science and Technology, Thuwal, Saudi Arabia

[3]Griffith Climate Change Response Program, Griffith University, Gold Coast Campus, QLD, 4222, Australia

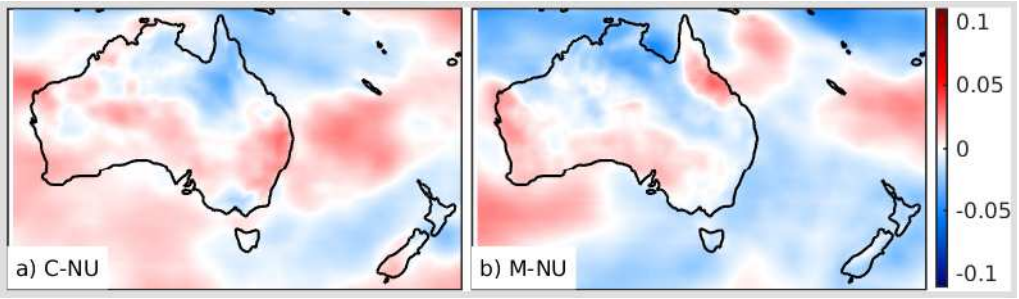

Figure S1: Neutral composites of WPD anomalies: The mean standardized anomalies of WPD corresponding to (a) C-NU and (b) M-NU. The color axis and the colorbar for Figure 2 and this figure are the same for easy comparison. The figure including the map and all the text elements has been plotted in MATLAB® version R2016a. (http://www.mathworks.com/products/matlab/).

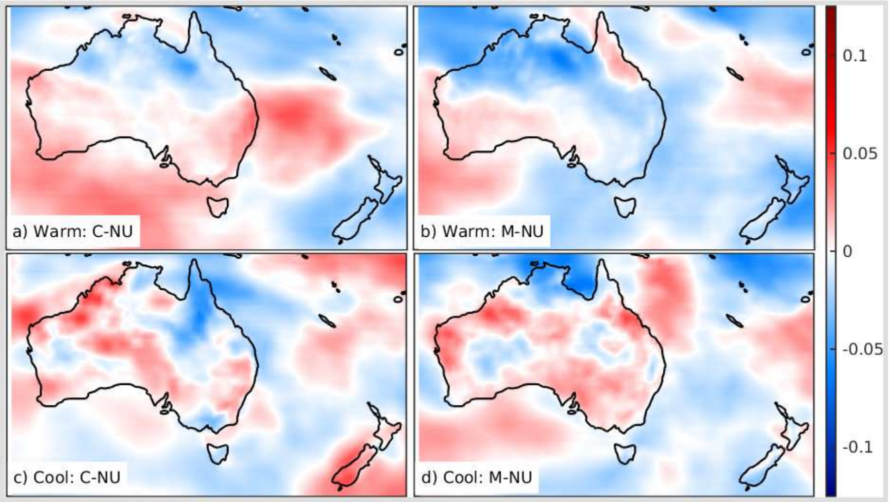

Figure S2: Seasonal neutral composites of WPD anomalies: The mean standardized anomalies of WPD corresponding to (a) C-NU in warm season, (b) M-NU in warm season, (c) C-NU in cool season, and (d) M-NU in cool season. The color axis and the colorbar for Figure 3 and this figure are the same for easy comparison. The figure including the map and all the text elements has been plotted in MATLAB® version R2016a. (http://www.mathworks.com/products/matlab/).
